# Composite CYP3A (CYP3A4 and CYP3A5) phenotypes and influences on tacrolimus dose adjusted concentration in adult heart transplant recipients

**DOI:** 10.21203/rs.3.rs-2921796/v1

**Published:** 2023-05-16

**Authors:** Savine Hernandez, Christina Aquilante, Kimberly Deininger, Joann Lindenfeld, Kelly Schlendorf, Sara Van Driest, Michelle Liu

**Affiliations:** CUAN; Vanderbilt University Medical Center

**Keywords:** tacrolimus, heart transplantation, immunosuppressive agents, pharmacokinetics, CYP3A4, CYP3A5, cytochrome P-450 CYP3A, genetics, pharmacogenetics

## Abstract

*CYP3A5* genetic variants are associated with tacrolimus metabolism. Controversy remains on whether *CYP3A4* increased [* *1B* (rs2740574), *1G (rs2242480)] and decreased function [*22 (rs35599367)] genetic variants provide additional information. This study aims to address whether tacrolimus dose-adjusted trough concentrations differ between combined CYP3A (CYP3A5 and CYP3A4) phenotype groups. Significant differences between CYP3A phenotype groups in tacrolimus dose-adjusted trough concentrations were found in the early postoperative period and continued to 6 months post-transplant. In CYP3A5 nonexpressers, carriers of CYP3A4*7Bor *7G variants (Group 3) compared to CYP3A4*1/*1 (Group 2) patients were found to have lower tacrolimus dose-adjusted trough concentrations at 2 months. In addition, significant differences were found among CYP3A phenotype groups in the dose at discharge and time to therapeutic range while time in therapeutic range was not significantly different. A combined CYP3A phenotype interpretation may provide more nuanced genotype-guided TAC dosing in heart transplant recipients.

## INTRODUCTION

A calcineurin inhibitor-based regimen is generally considered the cornerstone of immunosuppression following heart transplantation (HTx).^1^ Currently, the calcineurin inhibitor tacrolimus (TAC) is most often used; however, inter-individual variability and a narrow therapeutic window lead to challenges with both drug toxicities and efficacy.^2^ Despite frequent TAC monitoring and dose adjustment during the early postoperative period, many patients fail to maintain therapeutic TAC levels.^3,4^ Decreased time in the therapeutic range (TTR) has been associated with an increased risk of rejection, highlighting the importance of personalizing TAC dosing beyond standardized dosing and drug monitoring.^3–5^ One strategy that may improve patient outcomes is to use pharmacogenetic test results to optimize TAC dose and, ideally, attain a higher TTR.^6–8^ To effectively use this strategy, a better understanding of the impact of variants in two key pharmacokinetic genes on variability in TAC concentrations is needed.

TAC is metabolized in the liver and intestines by cytochrome P450 3A5 (CYP3A5) and 3A4 (CYP3A4) enzymes, and in solid organ transplant recipients *CYP3A5* variants can account for up to 45% of the variability in TAC dose requirements.^9,10^ Patients who are CYP3A5 expressers (*1 carriers) are predicted to have significantly lower dose-adjusted trough concentrations (C_0_/D) and require higher doses of TAC compared to patients who are CYP3A5 nonexpressers (*3*6, and *7homozygotes).^11^ Although *CYP3A5* variants are known to impact TAC pharmacokinetics, variants in *CYP3A4* may also play an important role, particularly for CYP3A5 nonexpressers.^11^ While there are no guidelines for CYP3A4-guided TAC dosing, *CYP3A4* variants predicted to increase enzyme function [*7B (currently known as c.−392G > A) and * *1G* (currently known as c.1026 + 12G > A)], and decrease enzyme function (*22) were associated with TAC metabolism in renal and lung transplant patients.^12–18^ Our current knowledge on the effect of the combined CYP3A phenotype on TAC concentrations in HTx recipients is limited.^8,19,20^ In addition, controversy remains on whether *CYP3A4* variants are clinically relevant in HTx recipients due to conflicting and limited literature as well as linkage disequilibrium between CYP3A5*3 and CYP3A4*1B (rs776746 and rs2740574) reported in some populations.^14,20^ A combined CYP3A (CYP3A4 and CYP3A5) phenotype can potentially provide a more nuanced metabolic profile and thus more accurately aid in individualizing TAC dosing post-transplantation.

To our knowledge no HTx study has evaluated CYP3A phenotypes using both CYP3A5(*3, *6, *7) and *CYP3A4* (*7B, *1G, *22) genetic variants. Thus, we sought to determine the effect of a predicted CYP3A composite phenotype on TAC pharmacokinetic outcomes in the HTx population. We hypothesize that CYP3A composite phenotypes influence TAC C_0_/D in HTx recipients in the early postoperative period and up to 6 months after HTx.

## METHODS

### Study design and population

We conducted a single-center, retrospective study using Vanderbilt University Medical Center’s (VUMC) BioVU, a DNA repository linked to de-identified electronic health records (EHR).^21,22^ Patients initially transplanted in the adult HTx program at VUMC were screened for inclusion. Patients were included if they received oral TAC postoperatively and had genotyping data available [CYP3A4 (*1B, *1G, *22) and CYP3A5(*3, *6, *7)]. Sublingual TAC doses were converted to oral equivalents (1:2 ratio) to account for differences in absorption.^23^

Patients were excluded if they received a combined HTx with another organ, were administered intravenous TAC, or had incomplete data (< 5 days of inpatient TAC data). TAC (dose and concentration), other medications (i.e. immunosuppressants, prophylactic antimicrobials, CYP3A inducers and inhibitors), and demographic data were abstracted from the EHR by manual review. Medication data were confirmed through inpatient administration data and manual review of notes. This study was reviewed and approved by the VUMC Institutional Review Board.

### Tacrolimus trough data

TAC monitoring and dose adjustments were performed per institutional protocol with no major changes throughout the study period. TAC trough concentrations (C_0_) were collected daily postoperatively during a patient’s index stay, and then at each subsequent HTx clinic visit thereafter. Dose adjustments were made by treating clinicians to achieve a TAC C_0_ of either 8–10 ng/ml or 10–12 ng/ml taking into account patient comorbidities, perceived rejection risks and potential drug-drug interactions. TAC C_0_ were retrospectively captured from the de-identified EHR. For the primary outcome analysis, missing troughs were estimated using the median of the previous and subsequent troughs.^18^ TAC concentrations measured <10 or >14 hours after the previous dose were excluded as non-true troughs.

### Outcome definitions

The primary outcome was median dose- and weight-adjusted TAC trough concentration (C_0_/D) from postoperative day (POD) 2 to discharge among CYP3A groups, capturing TAC pharmacokinetics in the inpatient setting. Post hoc analyses on CYP3A4 and CYP3A5 phenotypes were conducted to investigate impact of individual genes. C_0_/D from POD 14 to day 30 was also assessed to explore the early and late post HTx setting. The daily TAC C_0_/D was calculated as follows: C_0_/D = (TAC trough, ng/ml)/(TAC dose, mg/kg/d). The TAC dose was defined as the average of doses administered during the 48 hours (~ 4 half-lives) before the trough. If no doses were given in the preceding 48 hours (e.g. when doses were held due to elevated troughs), the daily C_0_/D was undefined and excluded. During a patient’s index admission, weight was defined as the recorded weight on the day of HTx or within 7 days prior when available. Following hospital discharge, weight was defined by weight on the day of discharge.

Additional pharmacokinetic outcomes of interest included median TAC C_0_/D at two, three-, and six-months posttransplant, TAC C_0_/D and dose at discharge, time in therapeutic range (TTR), time to therapeutic range (TtTR), percent of troughs in range, percent of subtherapeutic (≤ 8 ng/ml) and supratherapeutic levels (≥ 12 ng/ml) during POD 2 to 30. The Rosendaal linear interpolation method was used to calculate TTR using trough goal ≥ 8 and ≤ 12 ng/ml.^24^ The TtTR was assessed as the number of days between the first TAC dose and two consecutive troughs ≥ 8 and ≤ 12 ng/ml (where median of previous and post troughs were used for days of no trough levels).^18^

Given that moderate and strong CYP3A4 inducers and inhibitors are known to affect CYP3A, we conducted post hoc analysis to investigate the impact of concomitant drugs (identified by FDA and Flockhart tables) on the median TAC C_0_/D.^25,26^ We also assessed acute kidney injury (AKI) incidence and stage using the Kidney Disease Improving Global Outcomes guideline criteria from day of HTx to POD 30.^27^

### Genotyping and haplotype inference

We selected *CYP3A* single nucleotide variants (SNVs) previously associated with TAC dose and clinical response in solid organ transplant populations [CYP3A5*3 (rs776746), *6 (rs10264272), *7(rs41303343); CYP3A4*1B (rs2740574), *1G (rs2242480), and *22 (rs35599367, rs2740574)]. Patient genotypes were obtained from BioVU and previously assayed using the Illumina Expanded Multi-Ethnic Genotyping Array in the Vanderbilt Technologies for Advanced Genomics laboratory using manufacturer-specified reagents and protocols. All samples with ≥ 99% average call rates and passing quality control were included. CYP3A4*1G and CYP3A5*7 were imputed through statistical inference to the 1000G Phase3v5 reference panel using Minimac4 on the Michigan Imputation Server. Imputed variants had Rsq ≥ 0.97, indicating high-quality imputation.

CYP3A5 genotype and phenotype assignments were based on the Clinical Pharmacogenetics Implementation Consortium TAC guideline.^11^ CYP3A4 phenotype assignments and the four CYP3A composite phenotype groups were based on previous findings (Table 1).^11,18,19^ To our knowledge, no previous solid organ transplant studies have concomitantly examined *CYP3A4* increased and decreased function alleles in a combined phenotype and thus we are uncertain of the phenotype assignments for CYP3A4*1 B/*22 and *1G/*22, these individuals were assigned as CYP3A4 intermediate expressers. Given the uncertainty in a composite CYP3A phenotype functionality, groups 1 −4 (least to most predicted CYP3A activity) were used given no standardized terminology available to date.^11,18^

### Statistical analysis

Statistical analyses were performed using Stata software program Version 17 (Stata, TX). Hardy-Weinberg equilibrium (p < 0.001) was determined using the exact test. Shapiro-Wilk test of normality was used to determine the type of statistical test with median and interquartile range or mean and standard deviation reported for nonparametric and parametric statistics, respectively. Differences between groups were evaluated by Fisher’s exact tests for nominal or categorical data and Kruskal-Wallis or one-way ANOVA for continuous or ordinal variables. Post hoc analysis using Dunn’s test with Bonferroni correction was conducted for significant results following Kruskal-Wallis tests. A two-sided p-value at α ≤ 0.05 was considered statistically significant for all tests.

## RESULTS

### Study Population

**Figure 1:**
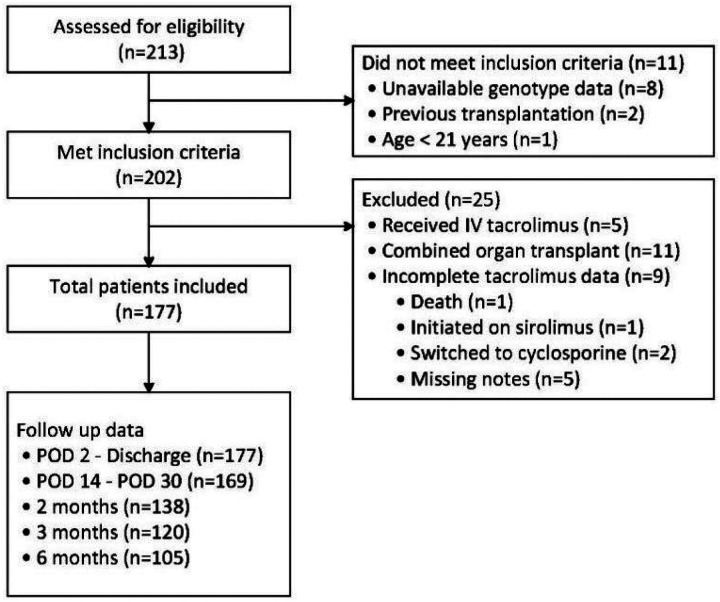
Study cohort flow diagram

In all, 213 patients were screened for eligibility, 36 of whom were eliminated by inclusion or exclusion criteria (Fig. 1). Thus, a total of 177 patients HTx between March 2008 and 2020 were assessed for the primary analysis with characteristics described in Table 2. The median age at transplantation was 54 years old [interquartile range (IQR) 45–61] with a median length of stay of 15 days (IQR 11 −24), and a mean baseline eGFR of 65.8 mL/min/1.72 m^2^. Most patients were male (68%), EHR self-identified as White (71 %) and had non-ischemic cardiomyopathy (68%) as their indication for HTx. Induction agents included antithymocyte globulin (39%), basiliximab (21 %), or high-dose methylprednisolone monotherapy (40%). Maintenance immunosuppression post-HTx included TAC, mycophenolate mofetil or mycophenolic acid, azathioprine (three patients switched from mycophenolate), and prednisone. TAC doses were predominately initiated at 1 mg twice daily [median (IQR) 0.1 mg/kg, (0.05–0.15)]. Prophylactic antimicrobial agents included trimethoprim-sulfamethoxazole, dapsone, or pentamidine for *Pneumocystis jirovecii* pneumonia prophylaxis, nystatin suspension or clotrimazole troches for fungal prophylaxis, as well as (val)ganciclovir and (val)acyclovir for viral prophylaxis.

### Observed genotypes and assigned phenotypes

Most patients (n = 82, 46%) were in CYP3A Group 2 as expected given the higher prevalence of self-identified White patients (Table 2). All genotype frequencies were in Hardy-Weinberg equilibrium (HWE), except for CYP3A4*1B, CYP3A4*1 G, and CYP3A5*3 (Table S1). When taking the population into consideration and analyzed separately, only CYP3A4*1B deviated from HWE in the White population. The diplotypes and allele frequencies are found in Table S2.

### Pharmacokinetic outcomes

#### CYP3A, CYP3A4 and CYP3A5 tacrolimus concentration/dose ratio

**Figure 2:**
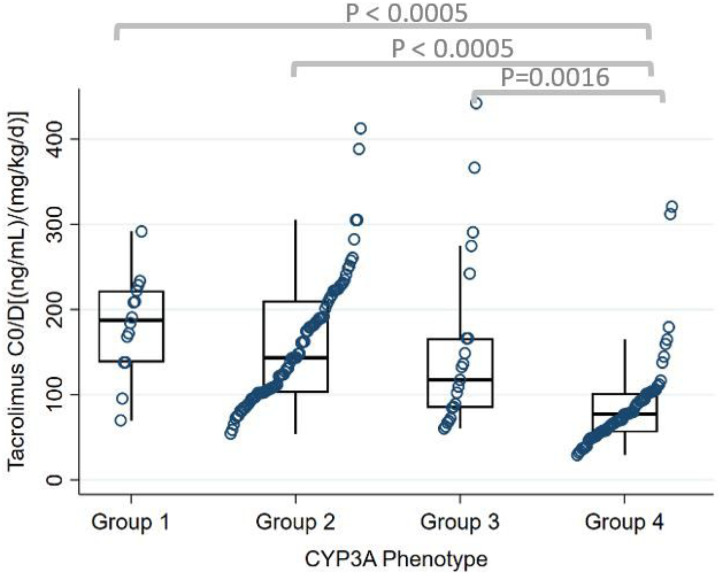
Tacrolimus C_0_/D from POD 2 to discharge by CYP3A phenotype

**Figure 3:**
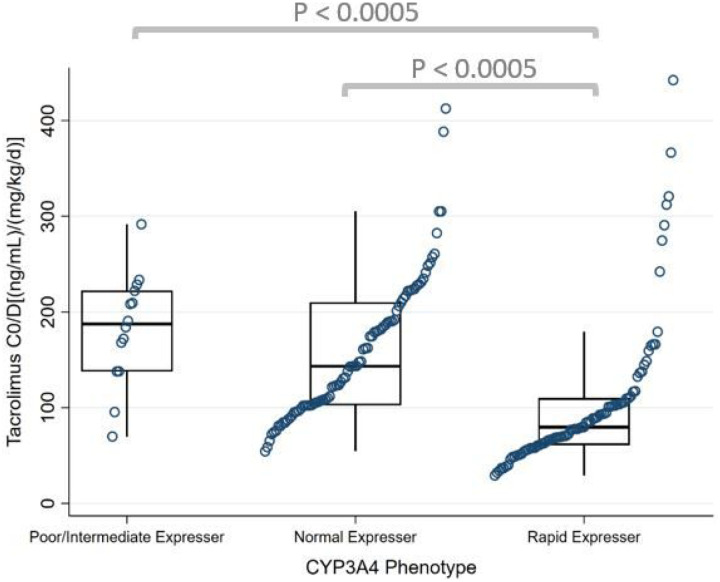
Analysis of tacrolimus C_0_/D from POD 2 to discharge by CYP3A4 phenotype

**Figure 4:**
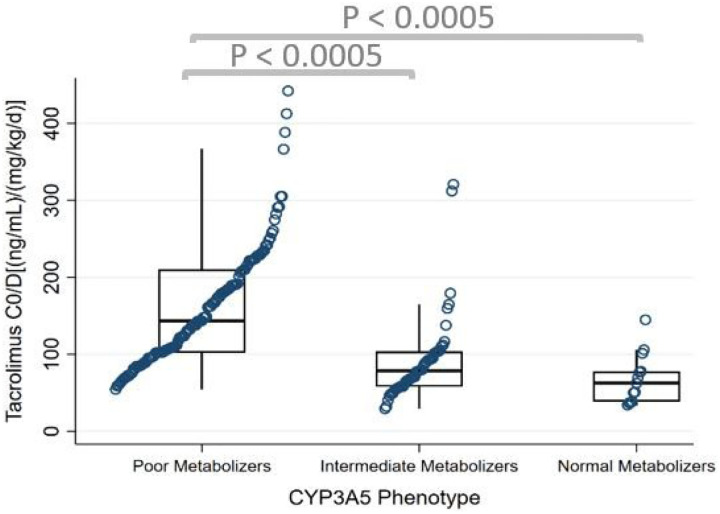
Analysis of tacrolimus C_0_/D from POD 2 to discharge by CYP3A5 phenotype

A total of 2,708 TAC troughs were included in the analysis. Median TAC C_0_/D from POD 2 to discharge for the whole cohort was 109 (ng/mL)/(mg/kg/d) [IQR 78–182]. There were statistically significant differences between the four CYP3A groups in median TAC C_0_/D from POD 2 to discharge (P = 0.001, Table 3). CYP3A Group 1 had the highest median TAC C_0_/D followed by Group 2, Group 3, and Group 4 (Fig. 2, Table 3). This difference was also sustained from POD 14 to 30 which had similar median TAC C_0_/D trends (P = 0.0001, Table S3). Group 4 had median TAC C_0_/D which was 59%, 46%, and 34% lower compared to Group 1, Group 2, and Group 3, respectively, during POD 2 to discharge. A similar pattern was found when the CYP3A4 and CYP3A5 phenotypes were analyzed separately in our post hoc analysis. CYP3A4 rapid expressers had 44% and 57% lower median TAC C_0_/D compared to normal expressers and poor/intermediate expressers, respectively, from POD 2 to discharge (P < 0.0005) (Fig. 3, Table S4). In the CYP3A5 phenotypes, intermediate and normal metabolizers had 45% and 56% lower C_0_/D compared to poor metabolizers, respectively, from POD 2 to discharge (P < 0.0005) (Fig. 4, Table S5).

#### Dose at discharge and ambulatory tacrolimus concentration/dose ratio

**Figure 5:**
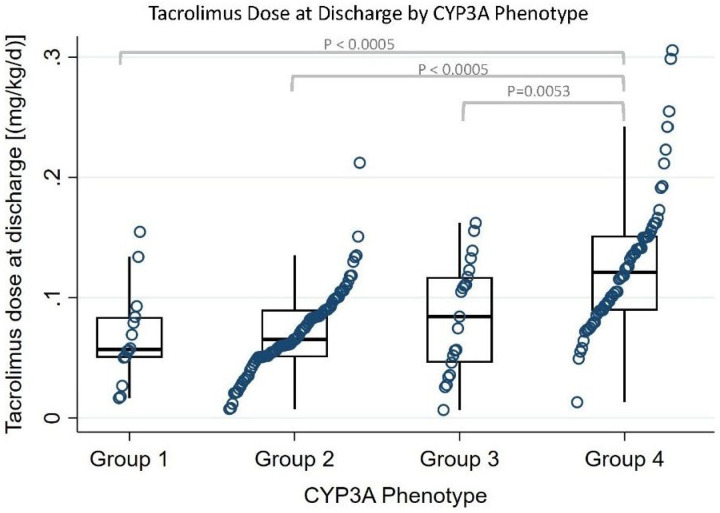
Tacrolimus dose at discharge by CYP3A phenotype

At discharge, CYP3A Group 1 had the highest and Group 4 had the lowest median TAC C_0_/D - at 2 and 6 months the trends were similar (Table 3). The opposite trend was seen for the dose at discharge where Group 4 had 2-, 1.7-, and 1.5-fold higher dose requirements compared to Group 1, Group 2, and Group 3, respectively (P < 0.0005, P < 0.0005, P = 0.0053, respectively, Fig. 5). At 2 months post-HTx, pairwise post hoc analysis showed the median TAC C_0_/D between Group 2 and Group 3 was significantly different (P = 0.02). Group 2 and Group 3 are CYP3A5 poor metabolizers, with the differences being that Group 3 are CYP3A4*1B or *1G carriers (rapid expressers) while Group 2 does not carry any *CYP3A4* variants. Additional outcomes for C_0_/D at discharge, 2 months, 3 months, and 6 months post-HTx are summarized in S4 and S5.

#### Time in therapeutic range, time to therapeutic range, and percent of therapeutic levels

TTR ranged from 30%−57% and the percent of troughs in therapeutic range ranged from 29–52% among the CYP3A groups; however, there were no significant differences across groups for either outcome (Table 3, Table S3). The TtTR was statistically different across groups (P = 0.008); CYP3A Group 1 took the fewest number of days, while Group 4 took the greatest number of days, to reach therapeutic range (7.5 vs 10.5 days, respectively, post-hoc P = 0.001). Outcomes for percent of subtherapeutic and supratherapeutic levels are summarized in Table S3. Drug-drug interactions and AKI outcomes analysis are available in the supplementary document (Table S6, Table S7).

## DISCUSSION

To our knowledge, this is the first study to evaluate the effect of combined CYP3A phenotypes in HTx recipients. We found differences in early post operative TAC C_0_/D between predicted CYP3A phenotype groups. In addition, these differences remained significant at 2, 3 and 6 months. We also found evidence suggesting differences in TAC C_0_/D are driven by *CYP3A4* variants in CYP3A5 nonexpressers, similar to a recent HTx study using *CYP3A5* genotyping and CYP3A4 phenotyping via mRNA expression.^20^ Our findings were evident at 2 months post-HTx, where a significant difference was observed in TAC C_0_/D between CYP3A Group 2 and Group 3 (P = 0.02). Given both groups are CYP3A5 nonexpressers, differences are likely attributable to CYP3A4*1B and * *1G* carrier status in CYP3A Group 3. However, differences driven by CYP3A4*22 in CYP3A5 nonexpressers (Group 1 vs Group 2) were not found potentially due to our small sample size (Group 1, n = 14) and the lack of CYP3A4*22 homozygous carriers in our cohort.

Prior to this study, combined CYP3A phenotypes with increased (CYP3A4*1B and *1G) and decreased (CYP3A4*22) function *CYP3A4* alleles had only been assessed in our previous study of lung transplant recipients.^18^ Similar to the lung transplant cohort, CYP3A4*22 and *CYP3A5*variant carriers (Group 1) in our HTx population had the highest TAC C_0_/D while CYP3A4 *1B,*1G variant carriers and *CYP3A5*expressers (Group 4) had the lowest TAC C_0_/D.^13,17,18^ Our previous work in lung transplant recipients showed that patients with the lowest predicted CYP3A activity (Group 1) had two-fold higher median C_0_/D and 51 % lower TAC dose requirements at discharge while patients with the highest CYP3A activity (Group 4) had 43% lower median C_0_/D and 1.7 fold higher dose requirements at discharge compared to patients who were *CYP3A5* nonexpressers and *CYP3A4* normal expressers (Group 2).^18^ In our HTx cohort, Group 1 had 1.3-fold higher median C_0_/D and 14% lower TAC dose requirements at discharge while Group 4 had 46% lower median C_0_/D and 1.7 fold higher dose requirements at discharge when compared to Group 2. The less variable difference between Group 1 and Group 2 in our HTx cohort compared to lung cohort suggests additional validation in differences between transplant populations.

Previously published transplant studies have investigated *in vivo* CYP3A4 activity as well as *CYP3A4* and CYP3A5 variants with increased or decreased function.^8,13,17,19,28^ Luo et al found endogenous CYP3A4 phenotype (assessed by urinary metabolic ratio) significantly correlated with TAC C_0_/D and weight-corrected daily dose in renal transplant recipients.^28^ After regression analysis, endogenous CYP3A4 phenotype, CYP3A5*3, and post-operative period accounted for 60% of the variability in C_0_/D. In CYP3A5 nonexpressers (CYP3A5*3/*3), CYP3A4 phenotype was responsible for 52% and 40% of the variability in TAC C_0_/D and dose, respectively; however, CYP3A4 phenotype accounted for only 15% and 11 % of the variability, respectively, among CYP3A5 expressers. Our study also supports TAC pharmacokinetic differences in *CYP3A4* variant carriers among CYP3A5 nonexpressers (Group 2 vs 3) at 2 months. Aouam et al. found that in renal transplant recipients, CYP3A4*1B carriers had significantly lower TAC C_0_/D and required higher doses to maintain therapeutic C_0_ compared to CYP3A4*1/*1.^17^ After a stepwise regression model, only *CYP3A4* polymorphism correlated significantly with C_0_/D variation regardless of the post-transplant phase and explained 22% of the variability in the late transplant phase (over 90 days). These renal studies show similar findings of *CYP3A4* variants accounting for significant C_0_/D and dose requirement variation.

In terms of CYP3A4*22, Deininger et al. evaluated CYP3A5and CYP3A4*22 in adult HTx recipients and found CYP3A4*22 was not significantly associated with TAC C_0_/D.^19^ Although the study found mean TAC C_0_/D was 1.8-fold lower in CYP3A “normal metabolizers” (CYP3A4*1/*1 + CYP3A5*1 carriers) compared to “intermediate metabolizers” (CYP3A4*1/*1 + CYP3A5*3/*3, or CYP3A4*22carriers + CYP3A5*1 carriers) and “poor metabolizers” (CYP3A4*22 carriers + CYP3A5*3/*3), the effect was largely found to be driven by CYP3A5*3. Our population’s frequency of CYP3A4*22 was similar (6.8% vs 6.6%), and we similarly did not find a significant difference in TAC C_0_/D between CYP3A4*22 in CYP3A5 nonexpressers (Group 2 vs Group 1). This suggests additional larger studies are needed to validate the clinical utility of CYP3A4*22 testing and to determine how results can be effectively incorporated with other *CYP3A* genetic results.

Regarding dosing, we found CYP3A Group 1 (CYP3A4*22 carriers) had the lowest TAC dose requirements at discharge. Gijsen et al. looked at pediatric HTx recipients and CYP3A4*22and found CYP3A poor expressers (CYP3A4*1/*22 and *CYP3A5* nonexpressers) required 48% less TAC than normal CYP3A metabolizers (CYP3A4*l/*1 and *CYP3A5-* expressers).^8^ Similarly, CYP3A Group 1 patients in our study required 50% (2-fold) lower weight-adjusted TAC doses than patients in Group 4 at discharge. Post hoc analysis showed Group 4 had statistically significant higher dose requirements compared to other Groups (2-, 1.7-, and 1.5-fold higher for Group 1, Group 2, and Group 3, respectively). Given that Groups 1 −3 are all CYP3A5 nonexpressers and the results show a gradient of decreasing dose requirements, our study suggests that CYP3A4 variants influence TAC dose requirements. Validating *CYP3A4* variants and the use of a combined CYP3A phenotype may provide granularity and better optimize TAC dose requirements for HTx patients, especially for those who are CYP3A5 nonexpressers where there currently is no recommended TAC dose adjustment.

We did not find any significant differences among CYP3A groups in TAC TTR using the Rosendaal method. This contrasts with our previous lung transplant study that found generally lower TTR (ranging 18–35%) in the cohort, with CYP3A Group 4 having the lowest precent TTR compared to the other groups (P = 0.03). This comparison should be made with caution, as the two studies used a different method of TTR calculation.^18^ However, in our HTx study we observed significant differences in TtTR with post hoc analysis showing that Group 4 consistently differed from Group 2 and Group 1. Although we cannot know if these differences are attributed to *CYP3A4* variants (as Group 4 are CYP3A5 expressers while Groups 1 and 2 are not), a TtTR difference of 3 days for Group 4 compared to Group 1 may be considered clinically meaningful and supports utilizing pharmacogenetic results to aid in faster optimization of Tac dosing.

### Limitations

Given the retrospective and single-centered nature, our study has limitations and is most applicable to populations similar to ours. Additional genetic factors, drug-drug interactions, as well as differences in oral formulations could also influence our findings. TAC is a substrate of P-glycoprotein (encoded by *ABCB1)* and influenced by P-glycoprotein inhibitors and inducers, which we did not assess given the conflicting evidence on utility of *ABCB1* variants.^18,29^ CYP3A4 rare and unknown function variants were not included in this study which may decrease accuracy of the phenotype prediction compared to comprehensive genotyping and phasing methods. Although we assessed for differences in TAC C_0_/D in patients who were concomitantly taking CYP3A inhibitors, there was a small sample size, therefore, our study is not powered to find differences pertaining to drug-drug-gene interactions. Patients were administered TAC capsules and suspension orally or through a nasogastric tube, which may affect the TAC pharmacokinetic profile. However, the relative bioavailability is found to be comparable and our outpatient results (where all patients were on capsules) were similar to those in the inpatient setting.^30^ Patient adherence and administration of TAC could not be verified in the outpatient setting; however, notes were reviewed to assess accuracy of doses. CYP3A4*1B likely deviated from HWE due to differences in allele frequencies in White populations.^11,23,24^ Lastly, there was a small sample size of CYP3A4*22 carriers (n = 14) and no homozygous carriers, limiting our analysis of the impact of CYP3A4*22 (CYP3A4 Group 1) on TAC C_0_/D.

In conclusion, a combined CYP3A phenotype significantly impacted TAC C_0_/D both in the early postoperative setting and up to 6 months following HTx. Consequently, dose requirements at discharge, and TtTR significantly differed between the groups. Post hoc analysis showed these differences were driven mainly by CYP3A4 Group 4 (CYP3A4*TB,*1G carriers) differing from other groups. Genotyping for both *CYP3A5* and *CYP3A4* variants can have the potential to provide more nuanced genotype guided TAC dosing to optimize patient outcomes. Further investigations are planned to assess the association of findings from this study with clinical outcomes including acute rejection, hospital readmission, development of de novo donor specific antibodies and graft vasculopathy, as well as to assess the effect of a combined CYP3A phenotype on patients of various ancestries. Our findings are most pertinent to populations similar to ours, enriched with CYP3A5*3 carriers. Future research in larger HTx populations across multiple centers are necessary to confirm our results and to assess the effect of a combined CYP3A phenotype on patients of diverse ancestries.

## Figures and Tables

**Table 1:** CYP3A Phenotype Assignments CYP3A phenotype assignment based on *CYP3A5* and *CYP3A4* allele functionality.

CYP3A Phenotypes	*CYP3A5*		
*CYP3A4* * ^,α,b^	*CYP3A5* expresser		*CYP3A5* nonexpresser
	Normal metabolizer (*1/*1)	Intermediate metabolizer (*1/*3, *1/*6, *1/*7)	Poor metabolizer (*3/3 *3/*6, *3/*7, *6/*6, *6/*7, *7/*7)
Rapid expresser (*1/*1B, *1B/*1B, *1/*1G, *1G/*1G)	CYP3A **Group 4**	CYP3A **Group 4**	CYP3A **Group 3**
Normal expresser (*1/*1)	Not observed	Not observed	CYP3A **Group 2**
Intermediate expresser (*1B/*22, *1G/*22)	Not observed	Not observed	CYP3A **Group 1**
Poor expressers (*1/*22, *22/*22)	Not observed	Not observed	CYP3A **Group 1**

CYP3A Groups 1–4: numbered least to most predicted CYP3A enzyme activity

* CYP3A4 Expresser: CYP3A4 phenotype groups determined based on limited previous literature available in contrast to established CYP3A5 phenotype groups^11,18^

α rs2242480, denoted as CYP3A4*1G in the manuscript has been recently changed to *c.1026 + 12G > A*

b rs2740574, denoted as CYP3A4*1B in the manuscript has been recently changed to *c.−392G > A*

**Table 2:** Baseline characteristics

Patient baseline characteristics	Total cohort (N = 177)
Age at transplantation, median (IQR), years	54 (45–61)
Male	121 (68.4)
Weight, mean (± SD), kg	87.4 (±17.9)
Body mass index, median (IQR), kg/m^2^	29.2 (24.9–33.2)
eGFR, mean (± SD), mL/min/1.73m^2^	65.8 (± 23.8)
Baseline serum creatinine, median (IQR), mg/dL	1.2 (1.0–1.5)
Ischemic time, median (IQR), min	163.5 (100–192)
Length of stay, median (IQR), days	15 (11–24)
**EHR-identified race**	
White	126 (71.2)
Black	48 (27.1)
Other	0
Unknown	3 (1.7)
**EHR-identified ethnicity**	
Non-Hispanic	172 (97.2)
Hispanic	0
Unknown	5 (2.8)
**CYP3A Phenotype**	
Group 1	14 (7.9)
Group 2	82 (46.3)
Group 3	21 (11.9)
Group 4	60 (33.9)
**Transplant indication**	
Non-ischemic cardiomyopathy	121 (68.4)
Ischemic cardiomyopathy	53 (29.9)
Congenital heart disease	3 (1.7)
**Induction Immunosuppression**	
Steroid only	71 (40.1)
Antithymocyte globulin	69 (39)
Basiliximab	37 (20.9)
**Maintenance Immunosuppression**	
Mycophenolate mofetil/mycophenolic acid	177 (100)
Azathioprine	3 (1.7)
Sirolimus	1 (0.6)
**Prophylactic agents**	
Sulfamethoxazole-trimethoprim	168 (94.9)
Dapsone	12 (6.8)
Pentamidine	1 (0.6)
Nystatin	176 (99.4)
Clotrimazole	5 (2.8)
Val(acyclovir)	160 (90.4)
Val(ganciclovir)	44 (24.9)

Abbreviations: IQR, Interquartile Range; SD, Standard Deviation; EHR, Electronic Health Record

**Table 3:** Primary and secondary pharmacokinetic outcomes for CYP3A

	CYP3A					
	Parameter (median, IQR)	Group 1	Group 2	Group 3	Group 4	P-value
Primary	C_0_/D POD2 - discharge [(ng/mL)/(mg/kg/d)]	187.36 (137.97–222.14)	143.45 (102.38–210.23)	117.35 (84.57–166.29)	77.00 (55.98–101.73)	0.0001
Secondary	Dose at discharge (mg/kg/d)	0.06 (0.05–0.08)	0.07 (0.05–0.09)	0.08 (0.05–0.12)	0.12 (0.09–0.15)	0.0001
	C_0_/D at discharge [(ng/mL)/(mg/kg/d)]	210.55 (121.56–236.57)	137.69 (105.74–191.15)	106.99 (80.57–202.09)	70.16 (56.26–114.64)	0.0001
	C_0_/D at 2 mo† [(ng/mL)/(mg/kg/d)]	165.81 (82.37–231.51)	155.65 (108.95–260.60)	91.26 (71.33–144.73)	71.05 (50.70–100.92)	0.0001
	C_0_/D at 3 mo† [(ng/mL)/(mg/kg/d)]	162.07 (132.02–182.83)	177.98 (109.97–280.49)	215.99 (132.01–265.36)	67.56 (51.20–110.02)	0.0001
	C_0_/D at 6 mo† [(ng/mL)/(mg/kg/d)]	262.89 (191.55–317.21)	162.31 (137.75–286.13)	157.67 (82.44–236.61)	72.75 (51.46–108.75)	0.0001
	Time in therapeutic range (%)	57.6 (28.3–61.2)	42.0 (29.5–57.7)	30.3 (20.5–48.1)	41.5 (23.0–57.4)	0.24
	Time to therapeutic range^α^ (days)	7.5 (6–8.5)	9 (7–11)	9 (8–11)	10.5 (9–15)	0.0008

CYP3A Groups 1–4: numbered least to most predicted CYP3A enzyme activity

† Patients without data in the outpatient setting could not be included in the analysis; 2 mo (n = 138), 3 mo (n = 120), and 6 mo (n = 105)

α Patients without two consecutive therapeutic tacrolimus concentrations could not be included in the analysis (n = 153)

## References

[1] McCormack PL, Keating GM. Tacrolimus: in heart transplant recipients. Drugs 2006; 66: 2269–2279; discussion 2280–2282. 17137409 10.2165/00003495-200666170-00010

[2] Söderlund C, Radegran G. Immunosuppressive therapies after heart transplantation--The balance between under-and over-immunosuppression. Transplant Rev (Orlando) 2015; 29: 181–189. 25812489 10.1016/j.trre.2015.02.005

[3] Adie SK, Bitar A, Konerman MC, Dorsch MP, Andrews CA, Pogue K Tacrolimus time in therapeutic range and long-term outcomes in heart transplant recipients. Pharmacotherapy 2022; 42: 106–111. 34882822 10.1002/phar.2653

[4] Baker WL, Steiger S, Martin S, Patel N, Radojevic J, Darsaklis K Association Between Time-in-Therapeutic Tacrolimus Range and Early Rejection After Heart Transplant. Pharmacotherapy 2019; 39: 609–613. 30892740 10.1002/phar.2262

[5] Sirota M, Heyrend C, Ou Z, Masotti S, Griffiths E, Molina K. Impact of tacrolimus variability on pediatric heart transplant outcomes. Pediatr Transplant 2021; 25: e14043. 34390091 10.1111/petr.14043

[6] Uno T, Wada K, Matsuda S, Terada Y, Oita A, Kawase A Impact of the CYP3A5*1 Allele on the Pharmacokinetics of Tacrolimus in Japanese Heart Transplant Patients. Eur J Drug Metab Pharmacokinet 2018; 43: 665–673. 29691732 10.1007/s13318-018-0478-6

[7] Deininger KM, Vu A, Page RL, Ambardekar AV, Lindenfeld J, Aquilante CL. CYP3A pharmacogenetics and tacrolimus disposition in adult heart transplant recipients. Clin Transplant 2016; 30: 1074–1081. 27314545 10.1111/ctr.12790

[8] Gijsen VMGJ, van Schaik RH, Elens L, Soldin OP, Soldin SJ, Koren G CYP3A4*22 and CYP3A combined genotypes both correlate with tacrolimus disposition in pediatric heart transplant recipients. Pharmacogenomics 2013; 14: 1027–1036. 23837477 10.2217/pgs.13.80

[9] Yu M, Liu M, Zhang W, Ming Y. Pharmacokinetics, Pharmacodynamics and Pharmacogenetics of Tacrolimus in Kidney Transplantation. Curr Drug Metab 2018; 19: 513–522. 29380698 10.2174/1389200219666180129151948 PMC6182932

[10] Haufroid V, Mourad M, Van Kerckhove V, Wawrzyniak J, De Meyer M, Eddour DC The effect of CYP3A5 and MDR1 (ABCB1) polymorphisms on cyclosporine and tacrolimus dose requirements and trough blood levels in stable renal transplant patients. Pharmacogenetics 2004; 14: 147–154. 15167702 10.1097/00008571-200403000-00002

[11] Birdwell KA, Decker B, Barbarino JM, Peterson JF, Stein CM, Sadee W Clinical Pharmacogenetics Implementation Consortium (CPIC) Guidelines for CYP3A5 Genotype and Tacrolimus Dosing. Clin Pharmacol Ther 2015; 98: 19–24. 25801146 10.1002/cpt.113 PMC4481158

[12] Li J, Liu S, Fu Q, Zhang Y, Wang X, Liu X Interactive effects of CYP3A4, CYP3A5, MDR1 and NR112 polymorphisms on tracrolimus trough concentrations in early postrenal transplant recipients. Pharmacogenomics 2015; 16: 1355–1365. 26228923 10.2217/pgs.15.78

[13] Zuo X, Ng CM, Barrett JS, Luo A, Zhang B, Deng C Effects of CYP3A4 and CYP3A5 polymorphisms on tacrolimus pharmacokinetics in Chinese adult renal transplant recipients: a population pharmacokinetic analysis. Pharmacogenet Genomics 2013; 23: 251–261. 23459029 10.1097/FPC.0b013e32835fcbb6

[14] Shi W-L, Tang H-L, Zhai S-D. Effects of the CYP3A4*1 B Genetic Polymorphism on the Pharmacokinetics of Tacrolimus in Adult Renal Transplant Recipients: A Meta-Analysis. PLoS One 2015; 10: e0127995. 26039043 10.1371/journal.pone.0127995 PMC4454552

[15] Pallet N, Jannot A-S, El Bahri M, Etienne I, Buchler M, de Ligny BH Kidney transplant recipients carrying the CYP3A4*22 allelic variant have reduced tacrolimus clearance and often reach supratherapeutic tacrolimus concentrations. Am J Transplant 2015; 15: 800–805. 25588704 10.1111/ajt.13059

[16] Bruckmueller H, Werk AN, Renders L, Feldkamp T, Tepel M, Borst C Which Genetic Determinants Should be Considered for Tacrolimus Dose Optimization in Kidney Transplantation? A Combined Analysis of Genes Affecting the CYP3A Locus. Ther Drug Monit 2015; 37: 288–295. 25271728 10.1097/FTD.0000000000000142

[17] Aouam K, Kolsi A, Kerkeni E, Ben Fredj N, Chaabane A, Monastiri K Influence of combined CYP3A4 and CYP3A5 single-nucleotide polymorphisms on tacrolimus exposure in kidney transplant recipients: a study according to the post-transplant phase. Pharma cogenomics 2015; 16: 2045–2054. 10.2217/pgs.15.138 26615671

[18] Liu M, Shaver CM, Birdwell KA, Heeney SA, Shaffer CM, Van Driest SL. Composite CYP3A phenotypes influence tacrolimus dose-adjusted concentration in lung transplant recipients. Pharmacogenet Genomics 2022; 32: 209–217. 35389944 10.1097/FPC.0000000000000472 PMC9177686

[19] Deininger KM, Vu A, Page RL, Ambardekar AV, Lindenfeld J, Aquilante CL. CYP3A pharmacogenetics and tacrolimus disposition in adult heart transplant recipients. Clin Transplant 2016; 30: 1074–1081. 27314545 10.1111/ctr.12790

[20] Déri M, Szakál-Tóth Z, Fekete F, Mangó K, Incze E, Minus A CYP3A-status is associated with blood concentration and dose-requirement of tacrolimus in heart transplant recipients. Sci Rep 2021; 11: 21389. 34725418 10.1038/s41598-021-00942-y PMC8560807

[21] Bowton E, Field JR, Wang S, Schildcrout JS, Van Driest SL, Delaney JT Biobanks and Electronic Medical Records: Enabling Cost-Effective Research. Sci Transl Med 2014; 6: 234cm3. 10.1126/scitranslmed.3008604 PMC4226414 24786321

[22] Roden DM, Pulley JM, Basford MA, Bernard GR, Clayton EW, Balser JR Development of a large-scale de-identified DNA biobank to enable personalized medicine. Clin Pharmacol Ther 2008; 84: 362–369. 18500243 10.1038/clpt.2008.89 PMC3763939

[23] Doligalski CT, Liu EC, Sammons CM, Silverman A, Logan AT. Sublingual administration of tacrolimus: current trends and available evidence. Pharmacotherapy 2014; 34: 1209–1219. 25251980 10.1002/phar.1492

[24] Rosendaal FR, Cannegieter SC, van der Meer FJ, Briët E. A method to determine the optimal intensity of oral anticoagulant therapy. Thromb Haemost 1993; 69: 236–239. 8470047

[25] Flockhart DA, Thacker D, McDonald C, Desta Z. The Flockhart Cytochrome P450 Drug-Drug Interaction Table. https://drug-interactions.medicine.iu.edu/MainTable.aspx (accessed 1 May2022).

[26] Center for Drug Evaluation and Research. Drug Development and Drug Interactions | Table of Substrates, Inhibitors and Inducers. FDA. 2022.https://wwwfda.gov/drugs/drug-interactions-labeling/drug-development-and-drug-interactions-table-substrates-inhibitors-and-inducers (accessed 12 Mar2023).

[27] Khwaja A. KDIGO clinical practice guidelines for acute kidney injury. Nephron Clin Pract 2012; 120: c179–184. 22890468 10.1159/000339789

[28] Luo X, Zhu L, Cai N, Zheng L, Cheng Z. Prediction of tacrolimus metabolism and dosage requirements based on CYP3A4 phenotype and CYP3A5 * 3 genotype in Chinese renal transplant recipients. Acta Pharmacologies Sinica 2016; 37: 555–560. 10.1038/aps.2015.163 PMC4820801 26924289

[29] van Gelder T. Drug interactions with tacrolimus. Drug Saf 2002; 25: 707–712. 12167066 10.2165/00002018-200225100-00003

[30] Undre N, Dickinson J. Relative bioavailability of single doses of prolonged-release tacrolimus administered as a suspension, orally or via a nasogastric tube, compared with intact capsules: a phase 1 study in healthy participants. BMJ Open 2017; 7: e012252. 10.1136/bmjopen-2016-012252 PMC5387971 28377389

